# State of the Art of Pharmacological Activators of p53 in Ocular Malignancies

**DOI:** 10.3390/cancers15143593

**Published:** 2023-07-12

**Authors:** Fabio Casciano, Enrico Zauli, Massimo Busin, Lorenzo Caruso, Saleh AlMesfer, Samar Al-Swailem, Giorgio Zauli, Angeli Christy Yu

**Affiliations:** 1 Department of Translational Medicine and LTTA Centre, University of Ferrara, 44121 Ferrara, Italy; 2 Department of Translational Medicine, University of Ferrara, 44121 Ferrara, Italy; 3 Department of Ophthalmology, Ospedali Privati Forlì “Villa Igea”, 47122 Forlì, Italy; 4 Istituto Internazionale per la Ricerca e Formazione in Oftalmologia (IRFO), 47122 Forlì, Italy; 5 Department of Environmental and Prevention Sciences, University of Ferrara, 44121 Ferrara, Italy; 6 Research Department, King Khaled Eye Specialistic Hospital, Riyadh 12329, Saudi Arabia

**Keywords:** p53 pathways, ocular malignancies, therapy, retinoblastoma, uveal melanoma, conjunctival melanoma, pterygium, eye cancer, tumor treatment

## Abstract

**Simple Summary:**

Ocular malignancies encompass a broad range of disorders that affect the eyelids, orbit, and eye and have significant impacts for national healthcare systems. Due to its exposure to various stressors, the eye is an anatomical site susceptible to cellular toxicity and tissue damage, which can result in significant vision loss. In this context, similar to other tissue types, p53 plays a crucial role in maintaining ocular homeostasis. However, few in vitro experimentation and clinical trials of p53 pathway modulators have been conducted. The aim of this review is to discuss the potential of pharmacological p53 activators as a novel targeted therapy for managing ocular tumors.

**Abstract:**

The pivotal role of p53 in the regulation of a vast array of cellular functions has been the subject of extensive research. The biological activity of p53 is not strictly limited to cell cycle arrest but also includes the regulation of homeostasis, DNA repair, apoptosis, and senescence. Thus, mutations in the p53 gene with loss of function represent one of the major mechanisms for cancer development. As expected, due to its key role, p53 is expressed throughout the human body including the eye. Specifically, altered p53 signaling pathways have been implicated in the development of conjunctival and corneal tumors, retinoblastoma, uveal melanoma, and intraocular melanoma. As non-selective cancer chemotherapies as well as ionizing radiation can be associated with either poor efficacy or dose-limiting toxicities in the eye, reconstitution of the p53 signaling pathway currently represents an attractive target for cancer therapy. The present review discusses the role of p53 in the pathogenesis of these ocular tumors and outlines the various pharmacological activators of p53 that are currently under investigation for the treatment of ocular malignancies.

## 1. Introduction

The eye is an anatomical site exposed to multiple stressors, such as microorganisms (viruses, bacteria, and protozoa), environmental factors (e.g., UV radiation, which leads to oxidative photodegradation), and degenerative disorders, which can cause moderate-to-severe vision loss and blindness.

Ocular diseases encompass various pathologies, including ocular malignancies and other complex eye diseases. The pathogenesis of various ocular tumors remains largely unknown, which limits the development of diagnostic and treatment approaches for these diseases that are relevant for health, society, and the national health system. Ocular malignancies may originate from any tissues of the eye, involving the conjunctiva, cornea, sclera, uvea, and/or retina with melanocytic, fibrous, epithelial, and other form of lesions [1]. In the United States and Northern Europe, the incidence of retinoblastoma is estimated to be approximately one in 16,000–18,000 live births [2,3]. On the other hand, melanoma is the most common malignant primary intraocular tumor in adults [4]. Resection and radiation therapy are the first-line treatment options for ocular melanoma, with enucleation being last resort [5].

Common eye disorders and diseases hold the potential to worsen the overall quality of life of patients and are commonly found in elderly people. These eye disorders include age-related macular degeneration (AMD), glaucoma, diabetic retinopathy, cataracts, and dry eye. These disorders are rising with an increase of blindness and vision loss patients from 12.44 million in 1990 to 22.56 million in 2019 [6].

In addition to diseases that mainly originate from tissues of the eye, paraneoplastic ocular syndrome may occur. In paraneoplastic ocular syndrome, molecular mimicry drives the autoantibodies response against normal eye tissue, leading to ophthalmic symptoms including, but not limited to, cancer-associated retinopathy, melanoma-associated retinopathy, cancer-associated cone dysfunction, paraneoplastic vitelliform maculopathy, and paraneoplastic optic neuritis [7].

In this context, the expression of p53 has been demonstrated throughout the human body, including the eye. The transcription factor p53 directly regulates the expression of target genes for maintaining tissue homeostasis during various physiological conditions such as development and differentiation [8,9,10]. Therefore, a mutation in the gene encoding p53 or the inactivation of the pathway under its governance may contribute to malignant transformation. In this regard, p53 preserves tissues by restricting genome alteration which may lead to aberrant mitoses [11]. Since the discovery that p53 plays a pivotal role in the induction of apoptotic cell death of DNA-damaged cells, the activation of p53 has become an attractive therapeutic strategy for various malignancies.

After the identification of compounds that modulate the p53 pathways, which arrest cell growth and induce apoptosis, multiple inhibitors of the p53–MDM2 interaction have been suggested as potential treatments for solid tumors, hematological cancers, and ocular diseases [12,13]. Alongside these inhibitors, recent research has identified traditional Chinese herbal medicine as a promising candidate for cancer therapy with fewer side effects than chemotherapy. Chinese herbal medicine may be considered as a complementary or alternative option in the field of oncology [14]. Natural products possess nutraceutical potential in a diverse range of diseases and are readily available with cost-effective pricing and fewer harmful side-effects [15,16,17,18]. Among these agents, baicalein, a flavonoid extracted from the dried root of *Scutellaria baicalensis*, has demonstrated significant efficacy in the treatment and prevention of many types of cancers. Baicalein exerts its actions by inhibiting numerous complex cascades and increasing the expression of the tumor suppression proteins p38 and p53, leading to cell cycle arrest and apoptosis [19].

The p53 network coordinates gene expression among cells and tissues, ensuring a central role in maintain organismal homeostasis [20].

Normal cells conserve pathways for maintaining genomic integrity, thus recognizing, and repairing damaged DNA. In response to certain types of DNA damage, the WRN gene, which encodes a DNA helicase, may activate p53 and potentiate p53-mediated apoptosis [21]. Indeed, as recently shown from Hao et al., the loss of WRN triggers DNA damage leading to the activation of p53/PUMA and subsequent cell death [22]. On the other hand, in progeria Werner’s syndrome, the ablation of the WNR gene may lead to the development of myeloid malignancies as result of competitive fitness by inactivating p53 [23]. Patients with Werner’s syndrome are also characterized by a high predisposition to various cancer types and as well as ocular cataracts [23,24]. The increased incidence of ocular cataracts in patients with Werner’s syndrome may be explained by the role of p53 in preventing cataracts and the existing relationship between WNR and p53. Indeed, single nucleotide polymorphisms (SNPs) of WRN might interfere with the binding of p53 to WNR, reducing the apoptotic function of p53 [25]. Furthermore, as shown by Reichel et al., p53 ablation increases the frequency of persistent hyperplastic primary vitreous and cataracts in a mouse model [26].

Therefore, given its role in tissue homeostasis, p53 is also widely expressed in the whole eye of an adult, where it plays a relevant role in maintaining ocular stability.

This review aims to address the involvement of p53 in ocular stability and highlight how the putative use of MDM2-p53 binding inhibitors, currently under clinical trial for other diseases, may improve ocular therapy, where the treatment methods might be the primary factor influencing the recurrence of the disease [27,28,29,30].

## 2. The Role of p53 in Tissue and Ocular Homeostasis

The p53 gene was first identified in 1979 as a 53 kDa protein associating with the middle T (mt) antigen of the SV40 virus [31]. Initially, it was believed to be an oncogene, due to its overexpression in SV40 transformed cells. Subsequent studies have shown it to be a humoral target in SV40-induced cancer model mice, and it was found to be expressed in 9% of patients with breast cancer [32,33]. In 1983, this protein was later named tumor suppression protein p53 [34], and finally further evidence from murine model leukemia and human leukemia cell line, wherein the gene encoding murine p53 protein was inactivated or deleted, suggested that p53 might be involved in tumor suppression [35,36].

Many pathways contribute to the p53 activation, including intracellular and extracellular stresses such as heat shock, UV light, inflammatory cytokines, oxidative stress, hypoxia, and mitogenic oncogenes, as well as physiologically cellular metabolic pathways observed during stem cell self-renewal and homeostasis. In response to these conditions, p53 is activated thereby promoting cell cycle arrest, DNA repair, senescence, and activation of the apoptotic pathway [37,38].

Under physiological conditions, the regulation of p53 depends on its post-translational protein turnover, which is mainly regulated by the murine double minus 2 (MDM2). MDM2 is primarily located at the nuclear level, by its intrinsic nuclear localization signal (NLS), and exerts the p53 nuclear export sequence to the cytoplasm, where proteasomal degradation can occur [39,40]. MDM2 acts as an E3 ligase that binds to the NH2 terminal domain of p53, targeting its ubiquitination and degradation by the 26S proteasome [41]. The E3 ubiquitin ligase activity of MDM2 relies on its interaction with the murine double minute X (MDMX also known as MDM4), forming an MDM2/MDMX complex with stable E3 ligase activity [42]. In mice models, targeted deletion of the MDM2 gene results in embryonic lethality due to p53-mediated apoptosis [43]. Therefore, MDM2 becomes a critical factor that transduces intrinsic and extrinsic signals to regulate the p53 effects in response to the perturbation of homeostasis [44].

It is well-known that p53 plays a role as a growth inhibitory factor and is incompatible with cancer cell proliferation. Indeed, p53 resides in the middle of the growth signals where growth-promoting conditions engage Akt which then mediates the phosphorylation of MDMX with the consequent stabilization of MDM2 [45]. This also demonstrates the pro-survival oncogenic activity of Akt and the cross talk between p53 and mTOR pathways [46,47]. These latter observations are in line with the alteration of the mTOR/AKT/PI3K pathway seen during diabetic retinopathy [48], the most common complication of diabetes mellitus (DM), a chronic metabolic disease characterized by hyperglycemia [49,50]. These findings support a putative relationship between p53 and the mTOR/AKT/PI3K pathway in the pathogenesis of diabetic retinopathy [48,51,52,53].

Activation of p53 relies on its intracellular increase via several mechanisms including the downregulation of p53 degradation, migration to the mitochondria and nucleus, and post-translational modification [54,55,56,57], which in turn suppress the interaction of p53 and MDM2 [58]. Once activated and phosphorylated at Ser15, p53 oligomerizes and forms tetramers that bind DNA to regulate transcription of target genes such as CDKN1A (p21, CIP1, WAF1), GADD45A13, pro-apoptotic genes such as NFRSF10B/TRAIL-R2, PUMA, and BAX, the pro-apoptotic Sept4/Apoptosis-related protein in the TGF-β signaling pathway (ARTS) gene, and interestingly also MDM2 [59,60,61,62,63,64,65,66]. Thus, the p53–MDM2 interaction is involved in a negative feedback loop that is important for p53 expression [67]. In addition, p21, which is a 21 kDa protein also known as WAF1, acts as a potent cyclin-dependent kinase 4 (CdK4)/cyclin D1 complex inhibitor essential for inducing cell cycle arrest mediating the downregulation of G1/S genes [68] and halting the cell cycle in the S and G2 phases in response to DNA damage [69]. Alternatively, activation of CdK4/Cyclin D1 leads to phosphorylation of the retinoblastoma protein (pRb) and cell cycle progression from G1 to S [70]. Many genes are under the p53-p21-pRb signaling pathway whereby DNA replication and repair processes are most prominently associated and in which p21 plays a crucial role in regulating pRb phosphorylation [71]. The resulting loss of function of the RB1 locus, following genetic mutation or deletion, leads to the activation of E2F family proteins, uncontrolled cell proliferation, and initiation of retinoblastoma [72,73].

Along with the nuclear migration and the regulation of the cell cycle, p53 may also promote the intrinsic apoptotic pathway, also known as mitochondrial apoptosis [63,74]. The intrinsic apoptotic pathway is driven by the Bcl-2 family of proteins, the activation of which depends on the p53 activity. This family includes many anti-apoptotic proteins (BCL-2, BCL-XL, BCL-W, MCL-1, and BFL-1/A1) and pro-apoptotic proteins (pore-formers BAX, BAK, and BOK as well as BH3-only BAD, BID, BIK, BIM, BMF, HRK, NOXA, and PUMA) [75]. The balance of these proteins determines the fate of the cell. The protein p53 exerts its apoptotic function through both direct and indirect protein–protein interactions. Once it has migrated to the nucleus, p53 may act as transcriptional activator, upregulating the expression of PUMA. PUMA acts as key a mediator of p53-driven apoptosis in two ways [76]: it directly mediates the inhibition of anti-apoptotic BCL-2, the inhibitor of cell death, thereby removing the direct the effect on other BCL-2 family protein, as well as by engaging and activating the pro-apoptotic BAX and BAK proteins [77,78]. The homo-oligomerization of BAX and BAK results in concomitant mitochondrial outer membrane permeabilization (MOMP) and the release of pro-apoptotic cytochrome c located in the mitochondrial membrane gap. Alternatively, p53 may mediate the mitochondrial release of cytochrome c through direct interaction in the cytosol with BAX [79].

**Figure 1:**
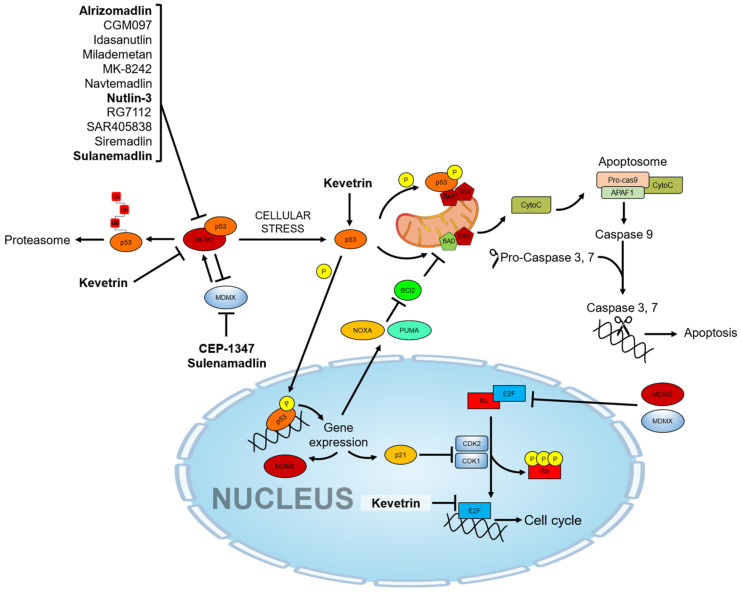
p53 molecular pathway and pharmacological activators. The drugs under investigation in ocular pathologies are highlighted in bold.

Cytochrome c can then activate a cascade of sequential activation of caspases by the formation of a complex so-called “apoptosome”, which contains cytochrome c, Apaf-1, and pro-caspase-9 [80]. Once caspase-9 is activated and associated with the apoptosome as a holoenzyme [81], it cleaves and activates downstream effector caspases as caspase-3 and caspase-7 [82]. Effector caspases are ultimately responsible for executing apoptosis by DNA fragmentation, subsequent cell shrinkage, and membrane blebbing (Figure 1).

Hypoxic stress also induces the accumulation of the p53 protein, which mediates the mitochondrial apoptotic pathway [83]. Conversely, most solid tumors contain regions with inadequate oxygen supply where the hypoxia-inducible factor (HIF), an oxygen-sensitive transcription factor, promotes tissue neovascularization aiding tumor cells to survive in the hypoxic environment, thereby contributing tumor growth. HIF-1α induces the protein phosphatase-1 nuclear-targeting subunit (PNUTS), which increases the proteasomal-dependent degradation of MDM2, indirectly rescuing p53 from the MDM2-mediated ubiquitination and leading it to p53-activation and mediated apoptosis [84]. In this cancer model, further evidence suggests that MDM2 also regulates angiogenesis by increasing the expression levels of transcription factors such as HIF-1α and vascular endothelial growth factor (VEGF), thereby promoting tissue neovascularization [85]. Hypoxia is a hallmark factor in the development of solid tumors as well as in the development of retinal diseases, including diabetic retinopathy (DR), age-related macular degeneration (AMD), and degeneration of retinal ganglion cells (RGCs) [48,86,87]. Among these, during retinal ischemia, hypoxic conditions invoke both p53 gene and protein expression, which in turn induce cell death [88,89]. This highlights that the pharmacological targeting of p53-related pathways may provide additional therapeutic benefits also to non-cancer ocular disease.

p53 is expressed at high levels during normal embryogenesis and development, regulating cell cycle and apoptosis [90], in the central nervous system (CNS) as well as other anatomical compartments such as olfactory bulb and eye [91,92,93,94,95,96]. Studies in an animal model showed a marked role of p53 during early embryonic ocular development, highlighting ocular abnormalities of hyaloid vasculature, vitreal opacities, retinal folding, and nerve fiber hypoplasia in mice defective for p53, according to their genetic background [97]. It is noteworthy that p53 exhibits widespread expression in various regions of the brain and the entire eye of adult mice. The expression is particularly high in the retina and optic nerve, while also accounting for a substantial portion, up to 70%, of the overall promoter activity expression in the cornea [98,99,100]. These observations are consistent with the high cytoplasmic expression of p53 in both the corneal and conjunctival epithelium of a typical murine eye, as well as the lack of its inhibitory modulator [98,101].

In this view, it is noteworthy that p53 is widely expressed in the normal eye, and it may play a role during ocular malignancies. The eye is an anatomic site exposed to multiple stressors such as microorganisms (viruses, bacteria, and protozoa), environments (UV radiation leads to oxidative photodegradation), and degenerative disorders all of which led to moderate-to-severe vision impairment and blindness [102,103,104]. Chronic inflammation resulting from the infectious disease may lead to the initiation of cancer, hampering growth regulators such as tumor suppressor p53 and affecting pathways of DNA repair with an accumulation of DNA damage [105]. Among these microorganisms, viruses have the capability to control physiological functions and pathways of host cells regulating growth arrest and apoptosis. Several studies have shown that human papillomavirus (HPV) is implicated in the development of pterygia and other related neoplasia of the ocular adnexa by the expression of viral oncoprotein that suppresses p53 activity [106,107,108]. However, Dushku et al. reported that HPV is not required as a cofactor in the development of pterygia and limbal tumors [109].

Intense light exposure can cause photochemical injury to the retina, ultimately leading to damage and apoptosis of retinal pigmented epithelial (RPE) cells, photoreceptors, and the entire neural retina [110,111,112]. The role of p53 in light-induced ocular degeneration may be mixed. As shown in the p53-/- mice model exposed to the intense blue light, apoptosis of photoreceptors may be both p53-independent [113,114], with the ocular degeneration mainly driven by the accumulation of the lipofuscin in response of the RPE cells to the light, and p53-dependent, with the upregulation of p53 and related genes leading to RPE cell death [115,116,117]. On the other hand, blue light clearly induces apoptosis of retinal Müller Cells [118].

Further, the eye is constantly subjected to oxidative stress due to its exposure to light, its high metabolic activity, and the oxygen-rich environment. Among those, UV radiation is the major source of reactive oxygen species, inducing a redox imbalance affecting various structures of the eye including the cornea, sclera, lens, and retina [119]. Under normal physiological conditions p53-induced processes cooperate to lower the ROS by promoting glutathione-dependent ROS scavenging and stimulating the expression of genes that reduce oxidative stress [120,121,122]. Consequently, oxidative stress also triggers oxidative DNA damage and cellular senescence, upregulating the p16INk4a/Rb and p53/p21Cip1 pathway and finally leading to cell cycle arrest [123]. In this light, oxidative stress and p53 become key factors in the development of eye-related diseases.

Therefore, p53 is widely expressed in the healthy human eye and plays an important role in the various function of eye tissues. Based on the relationship existing between p53/MDM2 in the ocular tissues, it appears that a therapeutic intervention with drugs that disrupt p53 inhibition by MDM2 and MDMX becomes interesting in the field of ocular diseases and merits pursuit.

## 3. Pharmacological Activators of the p53 Pathway

Several strategies can be used to target MDM2/MDMX for cancer therapy [124]. The currently available p53 cancer therapy relies on the interaction of p53 with its negative regulator MDM2, which can, in turn, be inhibited by the MDM2–p53 binding inhibitors including Nutlins. Nutlins (Nutlin-1, -2, and -3) are the first synthetic molecules developed by Hoffmann-La Roche in Nutley, based on 1,2,4,5-tetrasubstituted 4,5-cis-imidazolines, which interact with the p53-binding pocket of MDM2 and activate the p53 pathway, leading to cell proliferation arrest and/or apoptosis [125,126,127]. Nutlins are selective non-genotoxic inhibitors that do not induce the phosphorylation of p53 on Ser15 [65]. Among these, Nutlin-3 is a synthetic small molecule cis-imidazoline analog that mimics highly conserved hydrophobic amino acid residues including Phe19, Trp23, Leu22, and Leu26 within the hydrophobic pocket of MDM2 [126]. Nutlins have been shown to induce cell cycle arrest and cell death in a variety of solid tumors as well as in several types of hematological malignancies, viral infections, and cancer models with wild-type p53 including osteosarcoma, prostate cancer, Kaposi’s sarcoma-associated herpesvirus lymphomas, and neuroblastoma [128,129,130,131,132,133,134].

Since the discovery of Nutlins in 2004, several p53–MDM2 interaction inhibitors have been investigated for use in patients with various solid tumors and hematological cancers, as reported in Table 1.

**Figure 2:**
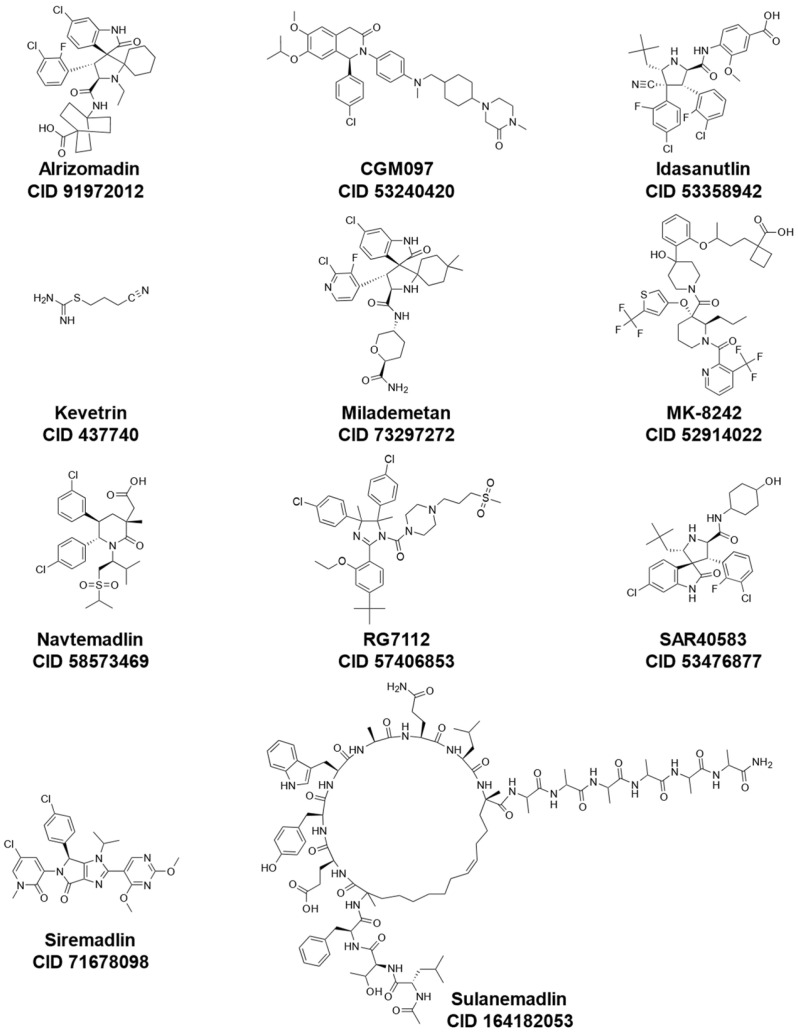
Chemical structures of representative p53-MDM2 interaction inhibitors cited in Table 2 (all obtained from PubChem; CID, CompoundID).

These inhibitors include Idasanutlin (also known as RG7388 or RO5503781) [135,136], RG7112 (also known as RO5045337) [137], Alrizomadlin (also known as APG-115) [138], SAR405838 (also known as MI-77301) [139], MK-8242 [140], Kevetrin [141], ALRN-6924 [142], Siremadlin (also known as HDM201) [143], Milademetan [144], CGM097 (also known as NVP-CGM097) [145], and AMG-232 (also known as KRT-232) [146]. In addition to small molecule inhibitors of MDM2, other compounds such as CEP-1347, originally developed for the treatment of other diseases (i.e., Parkinson’s Disease), have shown effectiveness in the activation of the p53 pathway [147]. An overview of mechanisms of actions, synonyms, molecular formulas, and chemical structures of these various drugs under investigation is shown in Table 2 and Figure 2.

## 4. Role of p53 Therapy in Pterygium

Although previously classified as a chronic degenerative condition, pterygium is now considered an uncontrolled cellular proliferation secondary to an abnormal expression of p53 protein within the conjunctival epithelium [148]. Histopathologically, pterygium is characterized by a focal fibrovascular proliferation of the conjunctival tissue with alterations in limbal stem cells, squamous metaplasia of conjunctival epithelium, dissolution of Bowman’s membrane, and excess proliferation of the stroma and extracellular matrix. Primary pterygium is locally invasive exhibiting tumorigenicity ranging from mild dysplasia to carcinoma in situ.

While the pathogenesis of pterygia remains poorly understood, the interaction between UV exposure and p53 mutations may likely play a role in its development and recurrence. Extensive data have demonstrated a dose-related relationship between chronic ultraviolet radiation and pterygium formation [149]. Similar to those seen in other skin cancer, UV irradiation may induce DNA damage in the p53 tumor suppressor gene including C to T transitions and CC to TT tandem mutations [150,151].

In a study by Spandidos et al., 60% of pterygia exhibited several DNA mutations including loss of heterozygosity and microsatellite instability, which result in alterations of the DNA repair pathways [152]. Through immunohistochemical analysis, mutant p53 protein has been found to be highly expressed in the epithelium overlying the pterygium [153,154,155,156]. Variability in p53 immunopositivity may reflect differences in race or environmental exposure [157]. The abnormal expression of p53 likely represents a failure in the regulation and control of the cell cycle caused by ultraviolet radiation, a well-known risk factor for pterygium formation [158]. Coupled expression of p53 and Bcl-2 is thought to result in a disruption of the transcriptional fidelity of p53 [159].

Surgical intervention is indicated in cases associated with high astigmatism, recurrent inflammation, or visual loss secondary to the involvement of the visual axis. However, following surgical excision, pterygium is associated with high recurrence rates ranging from 25% to up to 70% [156]. Thus, similar to the treatment of neoplastic diseases, the management of pterygium requires a multimodal approach including wide excision, antimetabolite chemotherapy, and irradiation. In order to overcome surgical infection, the intraoperative use of mitomycin C is commonly used. However, it is associated with several complications including necrotizing scleritis, scleral calcification, ulceration, damage to the corneal epithelium and endothelium, corneal edema, iritis, hypotony by injury of the ciliary body, glaucoma, and cataracts [160].

Activation of p53 by small-molecule antagonists of MDM2 can potentially induce apoptosis and regression in pterygium. Specifically, Nutlin has been proposed as a potential pharmacological treatment for pterygium. Indeed, experiments based on primary cell cultures, established from surgically excised specimens of primary pterygium, have demonstrated that treatment with Nutlin results in a 39-fold reduction in cell proliferation with dose-dependent inhibition of cell migration [161]. Additionally, no significant changes in cell viability and migration were observed in normal conjunctival cells treated with Nutlin. Further studies are required to evaluate the use of Nutlin and other MDM2 antagonists for the non-surgical treatment of pterygium.

## 5. Role of p53 Therapy in Conjunctival Melanoma

Conjunctival malignant melanoma is a rare, potentially life-threatening ocular malignancy that arises from the basal cells of the conjunctival epithelium [162]. Recognized risk factors for malignant conjunctival tumors include white race, older age, and exposure to ultraviolet light. Malignant transformation of primary acquired melanosis and, less commonly, conjunctival nevi has also been reported [163]. The presence of significant atypia associated with primary acquired melanosis is a significant prognostic indicator for malignant transformation.

Around 75% of conjunctival melanomas are believed to arise from primary acquired melanosis while approximately 5–10% arise from melanocytic nevi with atypia. In the remaining 5–10%, conjunctival melanoma arises de novo [164].

The typical presentation of conjunctival melanoma is a focal nodular melanotic epibulbar mass with multiple prominent feeder vessels extending to and from the lesion most commonly found at the limbus within the interpalpebral fissure. Some melanomas may be hypomelanotic or even amelanotic. Conjunctival melanomas have a strong propensity to metastasize to the preauricular or anterior cervical lymph nodes. Even with wide surgical excision and adjuvant chemotherapy, the overall response to treatment is poor with high rates of local recurrence and metastasis [165]. Adjuvant therapy may include topical mitomycin, plaque-brachytherapy, or proton-beam therapy. In advanced cases with lymphatic and hematogenous spread, enucleation and even orbital exenteration may be required [166]. Hence, novel therapeutic targets need to be explored to improve prognosis.

Immunohistopathology studies have shown that p53 is rarely expressed in conjunctival melanomas [167]. Although no clinical studies have been performed, the tumor suppressor p53 represents a potential therapeutic target for conjunctival melanoma. In conjunctival melanoma cell lines, treatment with Nutlin-3, a p53/MDM2 inhibitor, resulted in a time and dose-dependent decrease in cell viability with an increase in both MDM2 and p53. Compared to mitomycin C, stabilization of p53 and downregulation of IGF-1R were more effectively induced by Nutlin-3 [168].

## 6. Role of p53 Therapy for Retinoblastoma

Retinoblastoma is the most common neoplasm affecting the eye in children under five years of age [169]. The typical presenting signs include leukocoria and strabismus. As the tumor advances, affected individuals may develop hyphema, neovascular glaucoma, vitreous hemorrhage, or exudative retinal detachment. Extraocular tumor extension may be associated with significant proptosis and orbital inflammation [170].

While retinoblastoma represents a potentially life-threatening condition, current treatment modalities have significantly improved the prognosis, with disease-free survival rates approaching up to 100% [171]. However, the visual prognosis of retinoblastoma remains poor. In advanced disease, enucleation remains the standard of care. Although various strategies for globe salvage have been developed as alternative interventions, the rate of successful ocular salvage rate remains around 50–70%. Moreover, current chemotherapies are associated with toxicity resulting in various side effects. With an improved understanding of the tumorigenesis of retinoblastoma, novel adjuvant agents have been developed to target specific components and pathways. In particular, treatment using molecular genetics may individualize therapy based on specific tumor characteristics [172].

In retinoblastoma, various genetic changes result in tumor growth, proliferation, and metastasis. Consistent with the Knudson “two-hit hypothesis”, the development of retinoblastoma requires two mutational events in both alleles of the RB1 tumor suppression gene located in the long arm of chromosome 13 (13q14) [173]. Germline mutations of the RB1 gene result in dysfunctional pRb, which is a 928 amino acid phosphoprotein responsible for the regulation of gene transcription. In normal cells, pRb regulates progression through the cell cycle by interacting with the cellular E2F transcription factor and thereby blocking the transition from G1 to S phase [174]. Like the p53 family, alterations in the pRb function can result from multiple mechanisms including mutations in the RB1 gene itself and altered function of the promoter sequence [175]. In a normally healthy cell, loss of RB1 activity activates the transcription of p14ARF which in turn inactivates MDM2, leading to p53-mediated apoptosis and exit from the cell cycle [176,177]. On the other hand, MDM2 is an E3 ubiquitin ligase that mediates the interaction of Rb with the C8 subunit of the 20S proteasome, resulting in the ubiquitin-proteasome-mediated degradation of the RB1 tumor suppression protein [178]. MDM2-dependent degradation of Rb also increases DNA methyltransferase DNMT3A activity which is associated with the silencing of tumor suppressor genes [179]. Nonetheless, MDMX may inhibit RB protein degradation via MDM2 although it may contribute to the pRb degradation in a MDM2-dependent manner [180]. The pharmacological inhibition of MDMX by CEP1347 in wild-type p53 retinoblastoma cell lines, which overexpresses MDMX, leads to an increased p53 expression and activation of the p53 pathway [147]. Notably, greater than 70% of pediatric retinoblastoma patients demonstrate MDMX overexpression [181]. In this context, it is important to remember that MDMX also acts as a negative regulator by binding and sequestering p53 [182]. Elevated levels of MDM2 and MDMX proteins have been observed in certain cancers such as melanoma, Ewing’s sarcoma, and colon carcinoma [44].

Therefore, in the light of the interplay between pRb/E2F, MDM2/MDMX, and p53, the pharmacological modulator of p53-MDM2 interaction may be an attractive therapeutic target for retinoblastoma. Currently Nutlin-3, Kevetrin, ALRN-6924, and CEP-1347 are under investigation for their potential use in treating retinoblastoma.

Much attention has been given to the recently developed Nutlin class of MDM2 antagonists (Nutlin-1, -2, -3, and -3a) because of their non-genotoxic nature and potency in activating p53 [126,183,184]. By blocking the interaction between p53 and MDM2, Nutlin-3 releases p53 from negative control resulting in p53 stabilization and accumulation only in cells expressing wild-type p53 protein, thereby activating p53-dependent cell cycle arrest and apoptosis [185]. Nutlin-3 has been shown to suppress the proliferation of retinoblastoma cells both in vitro and in vivo. Co-immunoprecipitation experiments have demonstrated that Nutlin-3 not only binds MDM2 but also MDMX, although with much lower affinity [186]. In contrast to the p53-deficient retinoblastoma cell line (SJMRBL-8), retinoblastoma cells (Weri1) with wild-type p53 and MDMX overexpression were sensitive to Nutlin-3 [187]. In preclinical retinoblastoma models, the combined subconjunctival injection of Nutlin-3 with a topoisomerase inhibitor, topotecan, induced a p53 response that is similar to that induced by 5 Gy of ionizing radiation. The combined topotecan and Nutlin-3 improve the therapeutic index via a synergistic antineoplastic activity resulting in an 82-fold reduction in tumor burden without causing systemic or ocular adverse effects associated with prolonged exposure to broad-spectrum chemotherapeutic drugs [188].

Recently, the United States Food and Drug Administration has granted a rare disease designation to Kevetrin (thioureidobutyronitrile or 3-cyanopropyl carbamimidothioate hydrochloride). Kevetrin induces cell cycle arrest and apoptosis by altering the E3 ligase of MDM2, activating of the p53 gene, and increasing expression of p53-associated tumor suppressor proteins such as p21. Kevetrin also has therapeutic potential in advanced solid tumors of the ovary, lung, and breast. In a phase 1 clinical trial, patients with advanced solid tumors treated with Kevetrin exhibited a greater than 10% increase in p21 expression 7 to 24 h after treatment (NCT01664000). Additionally, Kevetrin potentially targets the altered Rb-E2F tumor suppressor pathway by downregulating E2F1, thus becoming a useful candidate for the treatment of this pathology [141]. Currently, Kevetrin has secured orphan drug status for ovarian cancer, pancreatic cancer, and retinoblastoma.

Moreover, ALRN-6924, a stabilized, cell-permeating peptide that inhibits both MDM2 and MDMX, is under investigation (NCT03654716) for use in retinoblastoma. ALRN-6924 has shown antitumor activity in phase I clinical trial for patients with lymphoma and solid tumors [142,189]. The mechanism of action of ALRN-6924 involves the inhibition of the interaction between p53 and MDM2 and MDMX, thereby inducing cell-cycle arrest or apoptosis in TP53-wild-type (WT) tumors.

Initially developed for Parkinson’s disease, CEP-1347 is a pharmacological inhibitor of MDMX that has also been shown to suppress the expression of MDM4 in retinoblastoma cell lines [147,190].

While several of these therapeutic agents are still under investigation, the future of retinoblastoma treatment will likely include these forms of anti-cancer therapy that target specific molecular genetic changes and aspects of the tumor microenvironment.

## 7. Role of p53 in Uveal Melanoma

Uveal melanoma is the most common primary intraocular tumor in adults. Approximately 85 percent of all ocular melanomas arise from the melanocytes of the uveal tract of the eye including the iris, ciliary body, and choroid [191]. The most common site for uveal melanoma is the choroid [192]. Although the development of uveal melanoma is largely considered sporadic, several risk factors including fair skin, light eyes, propensity to sunburn, and cutaneous nevi may predispose individuals to uveal melanoma [193]. Common symptoms of uveal melanoma include blurring of vision, photophobia, floaters, and visual field defects.

Uveal melanoma is a potentially fatal metastatic cancer. In approximately 50% of patients, uveal melanoma of the choroid and ciliary body spreads through the bloodstream to the liver, lung, bone, and skin [194]. Historically, enucleation was the only treatment option for uveal melanoma. Most patients are currently treated conservatively by means of plaque brachytherapy using iodine 125 or ruthenium 106 as an applicator [195]. Alternatively, patients undergo surgical resection, proton beam radiation therapy, or stereotactic radiosurgery using a cyber knife, gamma knife, or linear accelerator [196]. However, uveal melanoma is highly radioresistant and therefore requires treatment with high doses of radiation [194]. Moreover, radiotherapy is associated with several adverse events including radiation retinopathy, secondary glaucoma, and phthisis bulbi [197,198]. Even after successful radiation therapy, over 50% of patients with uveal melanoma eventually develop metastatic disease [199]. Thus, there has been increased interest in finding alternative therapy which results in a high tumor control rate and an improved safety profile.

In uveal melanoma, UV radiation may indirectly cause DNA damage through cytosine to thymine (C > T) transitions [200]. p53 serves as the main mediator of radiation-induced DNA damage [201], suggesting that uveal melanoma may be associated with functional defects that interfere with the p53 pathway [202], where gene mutations of TP53 are rare [203].

Defects within various downstream components of the p53 pathway, such as MDM2, could contribute to the relative radio resistance of uveal melanoma [204]. Analysis of the p53 pathway’s functionality has revealed defects within various downstream components, such as p21 and BAX [202]. Although this finding shows defects in the p53 pathway, Decaulin et al. have also demonstrated Bcl-2/XL/W and MDM2 co-inhibition as a promising target for treatment of uveal melanoma [205].

Hence, the restoration of p53 function by inhibiting its interaction with an MDM2 homolog represents a promising therapeutic strategy for this type of cancer. Consequently, the combination therapy with targeted agents and immunotherapy may further improve treatment response.

Recently, the United States Food and Drug Administration granted fast-track status to a phase 2 trial of the novel MDM2–p53 inhibitor alrizomadlin (APG-115), which demonstrated preliminary antitumor activity also in uveal melanoma [138]. Alrizomadlin is a selective, orally active, and potent spirooxindole-based small-molecule MDM2–p53 antagonist that destabilizes the MDM2–p53 complex and restores TP53 function [206]. By inhibiting the interaction between MDM2 and p53, alrizomadlin acts also as an immunomodulator and a regulator of a tumor’s immune escape mechanism, leading to enhanced T-cell mediated antitumor immunity. In various tumor models, alrizomadlin is a pharmacological p53 activator that has been found to promote an antitumor microenvironment, sensitize tumors that are resistant to PD-1 blockade, and enhance the efficacy of a PD-1 blockade independent of p53 status [207]. The combination of alrizomadlin and immune checkpoint PD-1 inhibitor enhances an antitumor response by reprogramming and downregulating of immunosuppressive M2 macrophages.

An open-label, sequential assignment, phase 1/2 clinical trial (NCT03611868) has evaluated alrizomadlin combined with pembrolizumab in patients with immunotherapy-resistant advanced solid tumors including melanoma, non–small cell lung cancer, STK-11–mutated lung adenocarcinoma, liposarcoma, urothelial carcinoma, and malignant peripheral nerve sheath tumors. Patients received 150 mg of alrizomadlin orally once every other day for two weeks, with one week off, and 200 mg of pembrolizumab was administered intravenously over 30 min on Day 1 of a 21-day cycle. Based on the preliminary and interim results of this study, patients in the uveal melanoma cohort who received alrizomadlin achieved an overall response rate of 14.3% due to one partial responder and a disease control rate of 71.4% with four cases of stable disease [208].

Interestingly, the p53 apoptosis effector related to PMP-22 (PERP) protein acts as the transcriptional target of p53 and has been found to influence tumorigenesis in uveal melanoma. Primarily localized in the plasma membrane, PERP is a tetraspan protein that stabilizes p53 through modulation of the interaction between p53 and MDM2. While its precise mechanism and function are currently unknown, PERP has been shown to induce p53-dependent apoptosis without resulting in cell cycle arrest [209]. Increased expression of PERP results in phosphorylation of the serine residues of p53, thereby disrupting the p53/MDM2 interaction between and enhancing pro-apoptotic gene transcription [210]. Preclinical studies have shown that PERP is a critical molecular determinant of apoptosis in primary uveal melanoma, where its downregulation is associated with aggressive disease. Therefore, PERP also represents a potential target for exploitation in enhancing p53 activity [210,211].

Currently, there are no approved targeted therapies for the treatment of early-stage ocular melanoma. However, the evolution of our understanding of the tumorigenesis and molecular characteristics of uveal melanoma has opened the possibility for targeted therapies.

## 8. Conclusions

Ocular tumors are a range of eye disorders that can cause moderate-to-severe vision loss, contributing to undesirable health outcomes. Degenerative disorders and causative agents (such as environmental and microbial factors) can prompt eye tissue alterations through the loss of intracellular processes that regulate cell cycle, DNA repair, and senescence and activate apoptotic pathways. The findings reported in this review suggest that the p53 pathway may be modulated in ocular disease and could represent a promising therapeutic target for ocular tumors. Although biological effects using ex vivo models have been demonstrated, only a few clinical trials of MDM2–p53 binding inhibitors for the treatment of ocular diseases have been conducted. Considering the multitude of effects driven by p53 activity in eye physiology, in vitro experimentation and clinical trials of these molecules could be undertaken to exploit the effects of p53 pathway activators for ocular disease treatment and develop novel targeted therapies for the management of ocular tumors.

## Figures and Tables

**Table 1:** p53–MDM2 interaction inhibitors under clinical trials.

Drug	Conditions	Phases, N	Status	NCT Number
**Idasanutlin (RG7388, RO5503781)**				
	PV	II, 27	Terminated	NCT03287245
	PV, ET	I, 13	Completed	NCT02407080
	AML	I/II, 24	Terminated	NCT03850535
	AML	I, 88	Completed	NCT02670044
	AML	III, 447	Terminated	NCT02545283
	AML, Acute Lymphoblastic Leukemia, Neuroblastoma	I/II, 183	Recruiting	NCT04029688
	AML	I, 122	Completed	NCT01773408
	NHL	I/II, 25	Terminated	NCT02624986
	NHL	I/II, 29	Terminated	NCT03135262
	Recurrent Plasma Cell Myeloma	I/II, 33	Active, not recruiting	NCT02633059
	CC	I/II, 94	Terminated	NCT03555149
	BC	I/II, 12	Terminated	NCT03566485
	ST	I, 8	Completed	NCT02828930
	ST	I, 48	Completed	NCT03362723
	ST	II, 770	Recruiting	NCT04589845
	GB	I/II, 350	Recruiting	NCT03158389
	ST	I, 99	Completed	NCT01462175
	ST	I, 61	Completed	NCT01901172
**Nutlin (RO5045337, RG7112)**				
	PV, ET	n.a., 131	Completed	NCT01970930
	AML	I, 43	Completed	NCT01635296
	Hematologic Cancers	I, 116	Completed	NCT00623870
	AML	I, 11	Completed	NCT01677780
	Sarcoma	I, 23	Completed	NCT01605526
	Sarcoma	I, 20	Completed	NCT01143740
	ST	I, 76	Completed	NCT01164033
	ST	I, 106	Completed	NCT00559533
**Alrizomadlin (APG-115)**				
	T-Prolymphocytic Leukemia	II, 36	Recruiting	NCT04496349
	AML, Chronic Myelomonocytic Leukemia, MDS	I/II, 69	Recruiting	NCT04358393
	AML, MDS	I, 102	Recruiting	NCT04275518
	Lymphoma, ST	I, 50	Completed	NCT02935907
	Neuroblastoma, ST	I, 100	Recruiting	NCT05701306
	Liposarcoma, ST	I/II, 92	Recruiting	NCT04785196
	***Uveal Melanoma, Melanoma, ST***	***I/II, 224***	***Recruiting***	NCT03611868
	Salivary Gland Cancer	I/II, 34	Recruiting	NCT03781986
**SAR405838 (MI-77301)**				
	ST	I, 26	Completed	NCT01985191
	ST	I, 77	Completed	NCT01636479
**MK-8242**				
	AML	I, 26	Terminated	NCT01451437
	ST	I, 48	Terminated	NCT01463696
**Kevetrin**				
	Ovarian Cancer	II, 2	Completed	NCT03042702
	ST	I, 48	Completed	NCT01664000
**Sulanemadlin (ALRN-6924)**				
	BC	I, 6	Terminated	NCT05622058
	AML, MDS	I, 55	Completed	NCT02909972
	BC, ST	I, 35	Active, not recruiting	NCT03725436
	***Retinoblastoma, Leukemia, Lymphoma, Brain Tumor, ST***	***I, 69***	***Active, not recruiting***	*** NCT03654716 ***
	Lymphoma, ST	I/II, 149	Completed	NCT02264613
	Lung Cancer	I, 35	Terminated	NCT04022876
**Siremadlin (HDM201)**				
	Hepatic Impairment	I, 48	Recruiting	NCT05599932
	AML, Allogeneic Stem Cell Transplantation	I/II, 38	Recruiting	NCT05447663
	Sarcoma	I/II, 58	Recruiting	NCT05180695
	AML	I, 2	Terminated	NCT04496999
	CC, ST	I, 24	Recruiting	NCT03714958
	Liposarcoma	I, 74	Completed	NCT02343172
	AML	I/II, 56	Recruiting	NCT05155709
	ST	I, 208	Completed	NCT02143635
	AML, MDS	I, 52	Active, not recruiting	NCT03940352
	AML	I/II, 0	Withdrawn	NCT03760445
	***Uveal Melanoma***	***I, 107***	***Terminated***	*** NCT02601378 ***
	Myelofibrosis	I/II, 45	Active, not recruiting	NCT04097821
	ST	II, 425	Recruiting	NCT04116541
	CC, Lung Cancer, BC, Renal Cell Carcinoma	I, 298	Completed	NCT02890069
**Milademetan (RAIN-32, DS-3032)**				
	AML	I, 14	Completed	NCT03671564
	AML	I, 10	Terminated	NCT03552029
	AML, Myelodysplastic Syndrome	I, 74	Terminated	NCT02319369
	AML	I/II, 21	Completed	NCT03634228
	Lymphoma, ST	I, 108	Completed	NCT01877382
	ST	II, 65	Recruiting	NCT05012397
	Liposarcoma	III, 160	Active, not recruiting	NCT04979442
**CGM097 (NVP-CGM097)**				
	ST	I, 51	Completed	NCT01760525
**Navtemadlin (AMG-232, KRT-232)**				
	AML	I/II, 18	Active, not recruiting	NCT04669067
	AML	I, 48	Suspended	NCT03041688
	AML	I, 36	Completed	NCT02016729
	AML	I, 24	Suspended	NCT04190550
	AML	I/II, 86	Recruiting	NCT04113616
	Chronic Myeloid Leukemia	I/II, 109	Recruiting	NCT04835584
	Chronic Lymphocytic Leukemia, NHL	I/II, 84	Recruiting	NCT04502394
	PM, PV, ET	I/II, 116	Recruiting	NCT04640532
	PM, PV, ET	II, 52	Recruiting	NCT04878003
	PM, PV, ET	II/III, 385	Recruiting	NCT03662126
	Myelofibrosis	I/II, 36	Recruiting	NCT04485260
	PV	II, 20	Unknown status	NCT03669965
	Plasma Cell Myeloma	I, 40	Recruiting	NCT03031730
	GB, Multiple Myeloma, ST	I, 107	Completed	NCT01723020
	Small cell Lung Cancer	II, 38	Recruiting	NCT05027867
	Non Small Lung Cancer	I/II, 92	Not yet recruiting	NCT05705466
	Merkel Cell Carcinoma	I/II, 115	Recruiting	NCT03787602
	GB, Gliosarcoma	I, 86	Suspended	NCT03107780
	Melanoma, ST	I, 31	Completed	NCT02110355
	Sarcoma	I, 46	Active, not recruiting	NCT03217266
	Endometrial Cancer	II/III, 268	Not yet recruiting	NCT05797831

Abbreviations: AML, Acute Myeloid Leukemia; BC, Breast Cancer; CC, Colorectal Cancer; ET, Essential Thrombocythemia; GB, Glioblastoma; MDS, Myelodysplastic Syndromes; n.a., not available; NHL, Non-Hodgkin’s Lymphoma; PV, Polycythemia Vera; PM, Primary Myelofibrosis. The drugs under investigation in ocular pathologies are highlighted in bold and italics.

**Table 2:** p53–MDM2 interaction inhibitors.

Drugs	Synonyms	Molecular Formula	Molecular Weight (g/mol)	Mechanism of Action
Alrizomadlin	APG-115	C_34_H_38_Cl_2_FN_3_O_4_	642.6	Blocks HDM2 interaction with p53
CGM097	NVP-CGM097	C_38_H_47_ClN_4_O_4_	659.3	Blocks HDM2 interaction with p53
Idasanutlin	RG7388, RO5503781	C_31_H_29_Cl_2_F_2_N_3_O_4_	616.5	Blocks MDM2 interaction with p53
Kevetrin	4-Isothioureidobutyronitrile, thioureidobutyronitrile	C_5_H_9_N_3_S	143.21	Induce activation of p53; Alters the E3 ligase processivity of MDM2; Induces p21 and PUMA
Milademetan	RAIN-32, DS-3032	C_30_H_34_Cl_2_FN_5_O_4_	618.5	Blocks MDM2 interaction with p53; Inhibits proteasome-mediated enzymatic degradation of p53; Restores p53 transcriptional activity and signaling
MK-8242		C_41_H_47_F_6_N_3_O_7_S	839.9	Blocks MDM2 interaction with p53; Restores p53 signaling
Navtemadlin	AMG-232, KRT-232	C_28_H_35_Cl_2_NO_5_S	568.6	Blocks MDM2 interaction with p53; Restores p53 transcriptional activity
RG7112	RO5045337	C_38_H_48_Cl_2_N_4_O_4_S	727.8	Blocks MDM2 interaction with p53; Stabilizes the p53 protein; Induces p53 target genes such as CDKN1A, NOXA, PUMA, Fas, and BAX
SAR405838	MI-77301	C_29_H_34_Cl_2_FN_3_O_3_	562.5	Blocks MDM2 interaction with p53; Inhibits proteasome-mediated enzymatic degradation of p53; Restores p53 transcriptional activity and signaling
Siremadlin	HDM201	C_26_H_24_Cl_2_N_6_O_4_	555.4	Blocks MDM2 interaction with p53; Inhibits proteasome-mediated enzymatic degradation of p53; Restores p53 transcriptional activity and signaling
Sulanemadlin	ALRN-6924	C_95_H_14_0N_20_O_23_	1930.2	Blocks HDM2 interaction with p53

Abbreviations: BAX, Bcl-2-associated X protein; CDKN1A, Cyclin-Dependent Kinase Inhibitor 1A; Fas, FS7-associated cell surface antigen; HDM2, human homolog of double minute 2; MDM2, mouse double minute 2 homolog; NOXA, Nuclear pOlypeptide eXpressed in Apoptosis; PUMA, p53 up-regulated modulator of apoptosis.
